# Comparative analysis of novel and conventional Hsp90 inhibitors on HIF activity and angiogenic potential in clear cell renal cell carcinoma: implications for clinical evaluation

**DOI:** 10.1186/1471-2407-11-520

**Published:** 2011-12-15

**Authors:** Jessica ES Bohonowych, Shuping Peng, Udhayakumar Gopal, Michael W Hance, Shane B Wing, Kelley M Argraves, Karen Lundgren, Jennifer S Isaacs

**Affiliations:** 1 Department of Cell and Molecular Pharmacology, Medical University of South Carolina, Charleston, SC, USA; 2 Cancer Research Institute, Central South University, Shanghai, China; 3 Department of Regenerative Medicine and Cell Biology, Medical University of South Carolina, Charleston, SC, USA; 4 Biogen Idec, San Diego, CA, USA

## Abstract

**Background:**

Perturbing Hsp90 chaperone function targets hypoxia inducible factor (HIF) function in a von Hippel-Lindau (VHL) independent manner, and represents an approach to combat the contribution of HIF to cell renal carcinoma (CCRCC) progression. However, clinical trials with the prototypic Hsp90 inhibitor 17-AAG have been unsuccessful in halting the progression of advanced CCRCC.

**Methods:**

Here we evaluated a novel next generation small molecule Hsp90 inhibitor, EC154, against HIF isoforms and HIF-driven molecular and functional endpoints. The effects of EC154 were compared to those of the prototypic Hsp90 inhibitor 17-AAG and the histone deacetylase (HDAC) inhibitor LBH589.

**Results:**

The findings indicate that EC154 is a potent inhibitor of HIF, effective at doses 10-fold lower than 17-AAG. While EC154, 17-AAG and the histone deacetylase (HDAC) inhibitor LBH589 impaired HIF transcriptional activity, CCRCC cell motility, and angiogenesis; these effects did not correlate with their ability to diminish HIF protein expression. Further, our results illustrate the complexity of HIF targeting, in that although these agents suppressed HIF transcripts with differential dynamics, these effects were not predictive of drug efficacy in other relevant assays.

**Conclusions:**

We provide evidence for EC154 targeting of HIF in CCRCC and for LBH589 acting as a suppressor of both HIF-1 and HIF-2 activity. We also demonstrate that 17-AAG and EC154, but not LBH589, can restore endothelial barrier function, highlighting a potentially new clinical application for Hsp90 inhibitors. Finally, given the discordance between HIF activity and protein expression, we conclude that HIF expression is not a reliable surrogate for HIF activity. Taken together, our findings emphasize the need to incorporate an integrated approach in evaluating Hsp90 inhibitors within the context of HIF suppression.

## Background

Hypoxia inducible factor (HIF) is a master regulator of the hypoxic response and plays a critical role in the development and progression of numerous solid cancers [1,2]. HIF functions as a heterodimeric transcription factor composed of an oxygen regulated α-subunit and a constitutively expressed β-subunit (or ARNT). HIF activity is tightly regulated by oxygen tension wherein its activity is restrained under oxygenated conditions via von-Hippel Lindau (VHL) ubiquitin ligase mediated degradation of the α subunit [3]. In contrast, tumor hypoxia facilitates HIF-α stabilization, dimerization, and transcriptional activation. HIF regulates a multitude of genes that contribute to pro-tumorigenic processes including invasion, angiogenesis and therapeutic resistance [2,4-6]. Importantly, inhibition of HIF function suppresses tumor formation and progression, and restores treatment sensitivity, highlighting HIF as a clinically relevant therapeutic target [1,7].

Clear cell renal cell carcinoma (CCRCC) tumors are highly vascularized and among the most lethal kidney tumors [8]. CCRCC, with its defined loss of VHL function and resulting constitutive HIF expression and activity, is a useful model to decipher the role of HIF in cancer progression and to evaluate HIF targeting strategies. Although the sufficiency of HIF for CCRCC remains somewhat controversial [9], HIF is a major participant in CCRCC within the context of VHL loss [10-13]. Of the two main pro-tumorigenic HIF-α isoforms, HIF-2α elicits tumor formation in CCRCC xenograft models [10,14] and appears to be more commonly upregulated in CCRCC relative to HIF-1α [4]. However, HIF-1α driven CCRCC xenograft models have also been documented [15], as well as compensatory mechanisms between the two isoforms [16]. Therefore, the targeting of both HIF isoforms may represent the most effective therapeutic approach. In spite of this, few studies have addressed the ability of candidate agents to target both isoforms.

A number of generalized HIF targeted approaches have been employed, including modulation of HIF expression, transcription, translation, dimerization, transactivation, and stability [17-23]. Small molecule inhibitors of the chaperone heat shock protein 90 (Hsp90) represent a growing class of clinically utilized anti-tumorigenic agents that have been collectively exploited as an alternative means of targeting HIF-α, given their shared ability to disrupt the ATP dependent chaperone activity of Hsp90 and block the protein folding of respective Hsp90 clients. HIF is an Hsp90 client protein [24] and we, and others, have shown that perturbing Hsp90 function with geldanamycin (GA) and small molecule derivatives promotes HIF-1α and HIF-2α protein degradation and suppression of transcriptional activity [25-27]. Importantly, Hsp90 targeted approaches bypass the requirement for both VHL and oxygen, instead utilizing the ubiquitin ligase RACK1 [25,28]. Therefore, these agents hold promise in tumor environments where VHL function is compromised, as in CCRCC or tumor hypoxia. In support of this premise, the Hsp90 inhibitors GA, 17-(allylamino)-17-demethoxygeldanamycin (17-AAG or Tanespimycin) and 17-dimethylaminoethylamino-17-demethoxygeldanamycin (17-DMAG or Alvespimycin) demonstrate anti-tumorigenic and anti-angiogenic properties in both *in vitro *and *in vivo *animal models, due in part to their ability to inhibit HIF function [29-33]. However, despite the promising pre-clinical actions of these inhibitors, clinical trials with 17-DMAG have been relatively unsuccessful for CCRCC and other solid tumors [34-36]. These failures highlight the critical need to further evaluate the effects of Hsp90 targeting agents upon HIF dependent signaling and angiogenesis in CCRCC and other cancers.

Since the advent of 17-AAG, numerous Hsp90 inhibitors exhibiting enhanced potency and diminished toxicity have been developed [37-41], leaving open the possibility that these next generation agents may demonstrate increased potency and efficacy *in vivo*. In addition to these direct Hsp90 inhibitors, histone deacetylase (HDAC) inhibitors, such as the clinically utilized LBH589 (Panobinostat), indirectly inhibit Hsp90 through protein hyperacetylation, leading to loss of chaperone function [42-44] and comparable inhibition of a subset of Hsp90 client proteins [45-47]. Similar to Hsp90 inhibitors, HDAC inhibitors ablate HIF levels and activity in several models [42,45,48-50]. Although one report examined the effects of HDAC inhibition upon HIF-1 expression in CCRCC cells [50] and another utilized LBH589 in combination with rapamycin [15], an analysis of these agents upon HIF-2 expression or activity is lacking, an important oversight given that a majority of CCRCC tumors preferentially express HIF-2 [4]. Moreover, no reports have directly compared the effects of ATP based competitive Hsp90 inhibitors with HDAC inhibitors such as LBH589 with respect to their respective ability to suppress the activity of HIF isoforms.

In this study, we utilized a prototypic next generation Hsp90 inhibitor, EC154, representing a novel synthetic compound with enhanced potency and diminished toxicity relative to 17-AAG. EC154 is a compound with improved properties (potency, pharmacokinetic and pharmacodynamic) over BIIB021, the first oral synthetic Hsp90 inhibitor to reach clinical trials [51]. EC154 binds to the Hsp90 ATP binding site in a manner similar to EC144 [52]. Towards the goal of identifying more potent HIF targeting agents, we evaluated the ability of 17-AAG, EC154, and LBH589 to suppress HIF activity and angiogenic potential in CCRCC cells. We show herein that although these agents exert inhibitory effects against HIF activity, they exhibited differential time and dose dependent responses not consistently linked to relative HIF protein expression. Our results therefore provide valuable guidance on the use of these agents as HIF inhibitors and highlight the biological complexity of Hsp90 inhibition upon suppression of HIF activity and downstream targets. Further, our results suggest that the evaluation of HIF expression in tissues as a surrogate for therapeutic efficacy may not suffice as a reliable indicator of HIF suppression.

## Methods

### Reagents and antibodies

The Hsp90 inhibitors 17-(Allylamino)-17-demethoxygeldanamycin (17-AAG) and EC154 were provided by the NCI Developmental Therapeutics Program and Biogen IDEC, respectively. The HDAC inhibitor LBH589 was provided by Dr. Peter Atajda (Novartis). HIF-1α (100-105) and HIF-2α (100-122) antibodies were from Novus Biologicals. Additional antibodies included GAPDH (9295) from Sigma; RACKI (17754) and topoisomerase II (13059) from Santa Cruz; P-ERK_T202/Y204 _(4370), ERK (4695), P-src_Y416 _(2101), src (2108) from Cell Signaling; and P-FAK_Y397 _(44-624G) and FAK (AHO-0502) from Invitrogen.

### Cell culture

UMRC2 and 786-O cells were provided by Dr. M.I. Lerman and Dr. R. Klausner (NCI, National Institutes of Health). UMRC2-VHL replaced cells were made as previously described [25] and 786-O VHL-replaced cells were provided by Dr. W.G. Kaelin (Dana-Farber Cancer Institute). All CCRCC cell lines were maintained in buffered Dulbecco's modified Eagle's medium (Thermo Scientific) supplemented with 10% fetal bovine serum (Gibco), L-glutamine (Thermo Scientific), and penicillin/streptomycin (Mediatech). Human umbilical vascular endothelial cells (HUVEC) were purchased from Invitrogen and maintained in Medium 200/LSGS (Gibco). All cells were maintained at 37°C and 5% CO_2_

### Western blots

Nuclear preparations were prepared as previously described [25]. Briefly, cells were washed in phosphate-buffered saline, collected using a hypotonic buffer (20 mM HEPES, 5 mM MgCl_2_, 5 mM NaCl, 1 mM EDTA, pH 7.9) containing protease inhibitors (Roche), nuclear pellets were collected following addition 10% Nonidet P-4, lysed in hypertonic buffer (20 mM HEPES, 400 mM NaCl, 1 mM EDTA) containing protease inhibitors, and supernatant collected. For whole cell lysates, cells were washed with phosphate-buffered saline, incubated in lysis buffer (20 mM Tris pH 7.5, 150 mM NaCl, 1%NP40, 1 mM EDTA) containing protease inhibitors, and supernatant collected. All protein concentrations were determined by BCA method (Thermo Scientific).

### qRT-PCR analysis

CCRCC cells were treated with 17-AAG, EC154, LBH589 or DMSO solvent control. Total RNA was extracted with the RNeasy Kit (Qiagen, Valencia, CA). Total RNA (1 μg) was used for reverse transcription with SuperScript^® ^III First-Strand Synthesis SuperMix for qRT-PCR (Invitrogen,) and resulting cDNA was used as a template for quantitative Real-time PCR analysis. The ratio of *GLUT1, VEGF, LOX1 *and *OCT-4 *to *α-tubulin *was measured with real-time-quantitative PCR (iQ5 Multicolor Real-Time PCR Detection System, Bio-Rad Laboratories, Hercules, CA) using iQ5 optical system software. The reaction mixture contained 12.5 μl SYBR green mix (Bio-Rad), 2 μl of mixed primers, and 2 μl cDNA template. The final volume was adjusted with H_2_O to 25 μl. Annealing temperature was 55°C for all reactions. Fluorescent products were measured by a single acquisition mode after each cycle. Primers are as follows: *α-tubulin*: F-AACGTCAAGACGGCCGTGT, R-GACAGAGGCAAACTGAGCAC; *GLUT1*: F-GTGGGCATGTGCTTCCAGTA, R-ACAGAACCAGGAGCACAGTGAA; *VEGF*: F-AGGCCAGCACATAGGAGAGA, R-TTTCCCTTTCCTCGAACTGA; *CAIX*: F-GGGTGTCATCTGGACTGTGTT, R-CTTCTGTGCTGCCTTCTCATC; qLOXI: F-ATGAGTTTAGCCACTTGTACCTGCTT, R-AAACTTGCTTTGTGGCCTTCA; *OCT-4*: F-GACAACAATGAGAACCTTCAG GAGA, R-CTGGCGCCGGTTACAGAACCA.

### Lentiviral infection and luciferase reporter assays

The Cignal Lenti-HIF Reporter system (SABiosciences) was used to stably transduce CCRCC cells with constitutively expressed renilla luciferase and HIF-regulated firefly luciferase constructs. UMRC2 and 786-0 cells were grown to 60-70% confluence and infected for 24 h according to the manufacturer's specifications. Cells were placed under puromycin selection and positive clones were expanded. For analysis of HIF activity, cells at 70% confluency were treated for 16 h with or without inhibitors, lysed, and dual luciferase activity analyzed (Promega). Experiments were performed in triplicate and arbitrary values normalized to renilla luciferase levels.

### Immunoassays

UMRC2 and 786-O cells were pre-treated for 4 h with the indicated compounds in low serum (3% FBS) medium. Cells were rinsed with PBS and then incubated with freshly prepared treatments in similar medium for 16 h under or normoxia or hypoxia (1% O_2_). Conditioned medium was collected following brief centrifugation and whole cell lysate was collected as described. VEGF and uPA concentrations were measured by ELISA (R&D Systems) according to the manufacturer's instructions, and normalized to total protein concentration of the conditioned medium.

### Tubule formation assay

HUVEC cells were serum starved overnight (0.1% M200) and plated (15,000 cells/well) in 96-well plates pre-coated with 50 μL of phenol-red free growth factor reduced Matrigel (BD Biosciences) with the indicated treatments. CCRCC conditioned medium was collected and added to HUVEC cells for 6 h. Effects on angiogenesis were determined by quantifying branch points in 6 replicate wells (1 field per well; 40X) for each treatment and are presented as percent of untreated control with standard deviation.

### Cell migration

Cell migration was assessed using Boyden chambers (0.8 μm; BD Biosciences). Cells were plated into the upper chamber in reduced serum DMEM (0.1%) with complete DMEM in the lower chamber and indicated agents added to both chambers. At 24 h, inserts were washed with PBS, the non-motile cells removed with cotton swabs, and the migratory cells fixed in formaldehyde, visualized with 0.1% crystal violet, and counted. The data presented represent the mean value from 4 replicates per treatment.

### Cell viability

CCRCC cells (5,000) were plated in 96-well plates, treated with 17-AAG, EC154, LBH589 or vehicle for 16 h and assessed for cell viability using the Cell-Titer Blue Reagent as per the manufacturer's instructions (Promega).

### Electrical Cell Substrate Impedance Sensing Assay (ECIS)

Changes in endothelial cell monolayer permeability were determined using the well established method of measuring electrical impendence [53]. HUVECs were seeded onto ECIS 8W10E+electrode arrays (Applied Biophysics, Troy, NY) precoated with human plasma fibronectin (Invitrogen) at 100 μg/mL in 0.15 M NaCl, 0.01 M Tris, pH 8.0. Transendothelial electrical resistance (TEER), an index of endothelial cell barrier function, was measured using an ECIS Model 1600 instrument (Applied Biophysics, Troy, NY). Cells were allowed to form a confluent monolayer and cell barrier until a plateau was reached (~3 days). In order to evaluate the ability of 17-AAG to reverse the effects of agents known to disrupt barrier function, culture medium was replaced with fresh EGM-2 containing the barrier disruptor, VEGF (50 ng/mL) in the absence or presence of 17-AAG (1 μM), and impedance was measured every 5 min at 15 kHz frequency. In order to evaluate the effects of CCRCC conditioned medium (CM) on endothelial cell barrier function, culture medium was replaced with CM collected for 24 h from 1 × 10^6 ^UMRC2 or 786-0 cells in the absence or presence of 17-AAG (1 μM), EC154 (100 nM), or LBH589 (100 nM). HUVEC-CM was used as a control for possible nutrient depletion. The traces shown represent the mean of duplicate wells for each treatment.

### Statistical analysis

Statistical significance was determined using one-way ANOVA followed by one-tailed student's T-tests. IC50 values were calculated using SigmaPlot10 (Systat) wherein curves were fitted to either a 3-parameter logistical or 4-parameter sigmoidal plot (R^2 ^= 0.695-0.996).

## Results

### Differential effects of inhibitors upon HIF protein expression are isoform and cell context dependent

**Figure 1:**
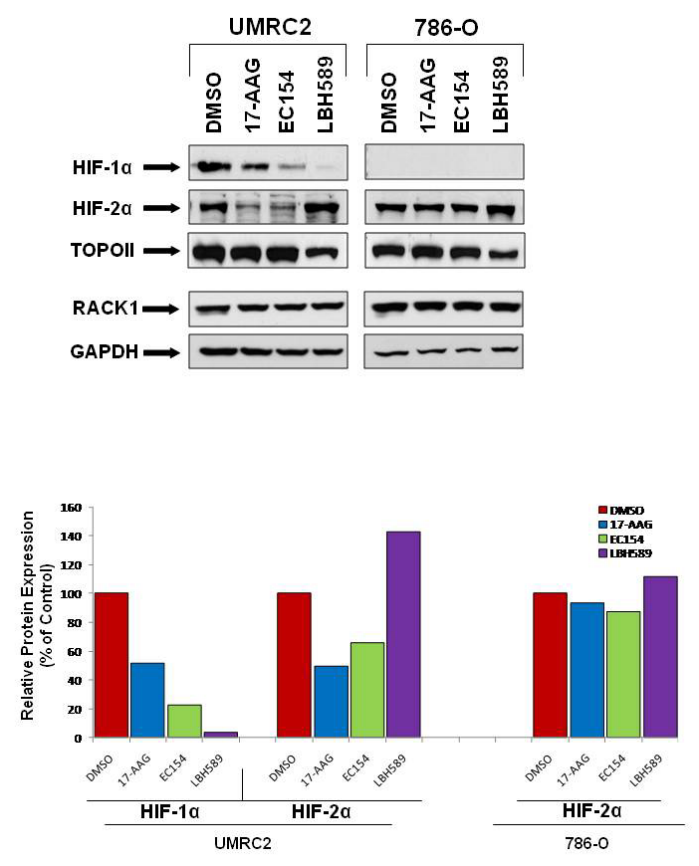
**HIF-1α and HIF-2α expression is differentially modulated by Hsp90 inhibition in CCRCC cells**. UMRC2 and 786-O cells were treated for 20 h with the Hsp90 inhibitors 17-AAG (1 μM), EC154 (100 nM), the HDAC inhibitor LBH589 (100 nM), or DMSO vehicle control. Nuclear protein was analyzed for HIF-1α and HIF-2α and cytosolic protein was analyzed for RACK1 by SDS-PAGE and Western blot. Topoisomerase II and GAPDH were used as nuclear and cytosolic loading controls, respectively. Bands corresponding to HIF-1α and HIF-2α Western blots were quantified, normalized to TOPOII, and are expressed as a percentage of control samples.

Given the prominent tumorigenic role of HIF in the majority of solid tumors, it is critically important to include evaluation of this metric when evaluating the efficacy of novel next generation Hsp90 inhibitors. To begin to address this question, we performed a comprehensive evaluation of the effects of 17-AAG, EC154, and LBH589 upon cellular HIF-1α and HIF-2α protein expression and activity in UMRC2 cells which express both HIF-1α and HIF-2α proteins and 786-O cells, which express only the HIF-2α isoform [54,55]. All three agents reduced HIF-1α expression in UMRC2, with EC154 more effective than 17-AAG, and LBH589 almost completely eliminating HIF-1α expression (Figure 1). Interestingly, the HIF-2α expression in 786-O cells was refractory to these treatments. Equally unexpectedly, LBH589 elicited a reproducible increase in HIF-2α expression in both 786-O and UMRC2 cells. In contrast to the effects in 786-O, 17-AAG and EC154 reduced HIF-2α expression in UMRC2, indicating that the effects of these drugs upon HIF isoforms may be cell context dependent. It has been shown that the VHL independent Hsp90 mediated destruction pathway for HIF-1α utilizes the adaptor protein RACK1 [28]. Given the apparent insensitivity of HIF-2α protein in response to Hsp90 inhibition, we examined the relative levels of RACK1 in these cell lines. As shown in Figure 1, RACK1 expression was abundant in both cell lines and was unaffected by drug treatment, indicating that sufficient RACK1 was available to mediate HIF-α degradation. Therefore, the mechanism for the sustained HIF-2α protein stability in 786-O cells following Hsp90 inhibition remains to be further clarified.

### Comparative effects of inhibitors upon HIF transcriptional activity

**Figure 2:**
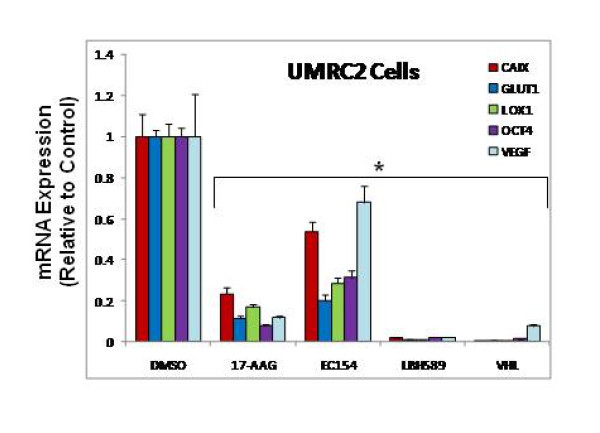
**The inhibitors 17-AAG, EC154, and LBH589 inhibit HIF-dependent gene transcription in CCRCC cells**. The CCRCC cell line UMRC2 was treated for 16 h with inhibitors as in Figure 1. Total mRNA was isolated and HIF-α regulated genes analyzed by qRT-PCR. Values were normalized to GAPDH and are presented relative to control cells, with standard deviation shown. All drug treatments significantly reduced all transcript levels (*) as determined by ANOVA and Student's *t-*test (*p *< 0.05).

**Figure 3:**
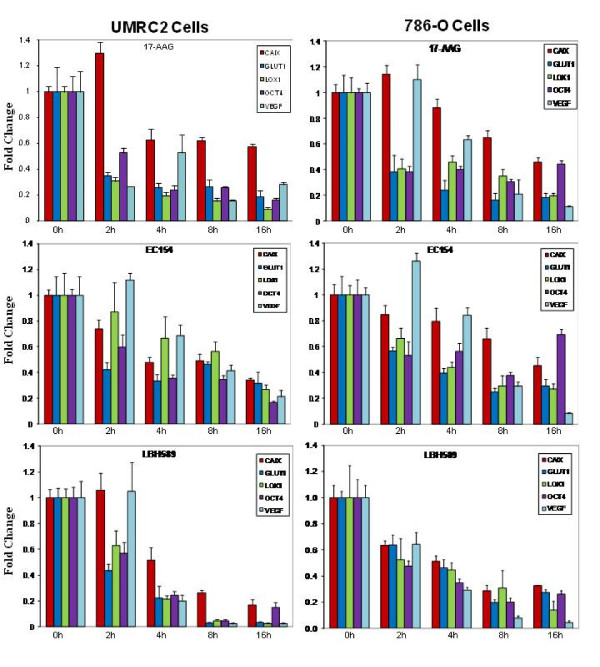
**Time dependent evaluation of 17-AAG, EC154, and LBH589 upon HIF-dependent gene transcription**. The CCRCC cell lines UMRC2 and 786-O were treated as in Figure 2 for the indicated times, total mRNA isolated and HIF-α regulated genes analyzed by qRT-PCR. Values were normalized to GAPDH and are presented relative to values at time zero with standard deviation shown.

To correlate HIF protein expression with HIF activity following drug exposure, we next evaluated HIF target gene levels by qRT-PCR. Dose curves at 16 h treatments were used to determine the most efficacious concentration for each drug (Additional file 1: Figure S1), and these optimal concentrations were utilized for all further studies (1 μM 17-AAG, 100 nM EC154, and 100 nM LBH589). VHL replaced derivatives were included as a relative negative control for HIF activity. The co-expression of both HIF-α isoforms in UMRC2 cells [55], coupled with the potential ability of the suppression of one HIF isoform to modulate expression and activity of the remaining protein [56-58], present challenges in evaluating the effects of these inhibitors upon HIF-1 and HIF-2 function. To address this question, we selected five transcripts, two of which are preferentially regulated by HIF-1α (*CAIX, LOX1*), two by HIF-2α (*GLUT1, OCT-4*), and one influenced by both isoforms (*VEGF*). As shown in Figure 2, all three drugs significantly inhibited mRNA expression of the selected transcripts in UMRC2, with LBH589 exhibiting the most profound suppression, comparable to VHL replaced cells. This result also indicates that the LBH-589 mediated increase in HIF-2α expression did not elicit a comparable increase in HIF-2α activity. Although *CAIX *and *VEGF *appeared to be more resistant to suppression in EC154 treated cells, this trend was not observed in subsequent experiments (Figure 3). The effect of these agents in 786-O was generally more modest when compared to UMRC2, although the general trends were mirrored, with 17-AAG demonstrating enhanced suppression of HIF driven transcripts when compared with EC154 (Additional file 1: Figure S1). Although *CAIX *and *LOX-1 *are reported to be controlled by HIF-1 activity [59,60], the apparent suppression of these transcripts following drug treatments in 786-O indicates that HIF-2α may also influence their transcriptional regulation.

The clinical administration of therapeutic agents results in a dynamic response largely influenced by concentration flux and gradients within the tumor. Our data in Figure 2 indicate that HIF targets exhibit differential sensitivity to these agents, a finding that may profoundly influence *in vivo *drug efficacy and contribute to dynamic responses. To more carefully analyze this parameter, we examined the time-dependent effects of these agents over a period of 2-16 h. As shown in Figure 3, all three inhibitors elicited a time dependent suppression of HIF regulated transcripts, as well as variable kinetics. *CAIX *transcript levels were more refractory to suppression by 17-AAG in both cell lines at earlier time points, while the remaining HIF transcripts in UMRC2 cells demonstrated a rapid and maximal suppression by 2 h. In 786-O cells, *VEGF *message was refractory to inhibition at earlier time points. With EC154 treatment, *CAIX *exhibited earlier suppression, while *VEGF *message was refractory at earlier time points in both cell lines. Although EC154 mediated suppression of transcripts occurred with slower kinetics, the overall extent of suppression at later time points was comparable to that observed with 17-AAG. Interestingly, LBH589 demonstrated the most potent suppression of transcripts in UMRC2, while 786-O transcripts were more refractory by comparison. Part of the complexity observed with HIF driven transcripts may be due to additional signaling pathways and effectors that contribute to their regulation [61-63] as supported by the differential expression of these transcripts in VHL replaced 786-O cells (Additional file 1: Figure S1).

### Comparative effects of inhibitors upon VEGF promoter activity and VEGF secretion

**Figure 4:**
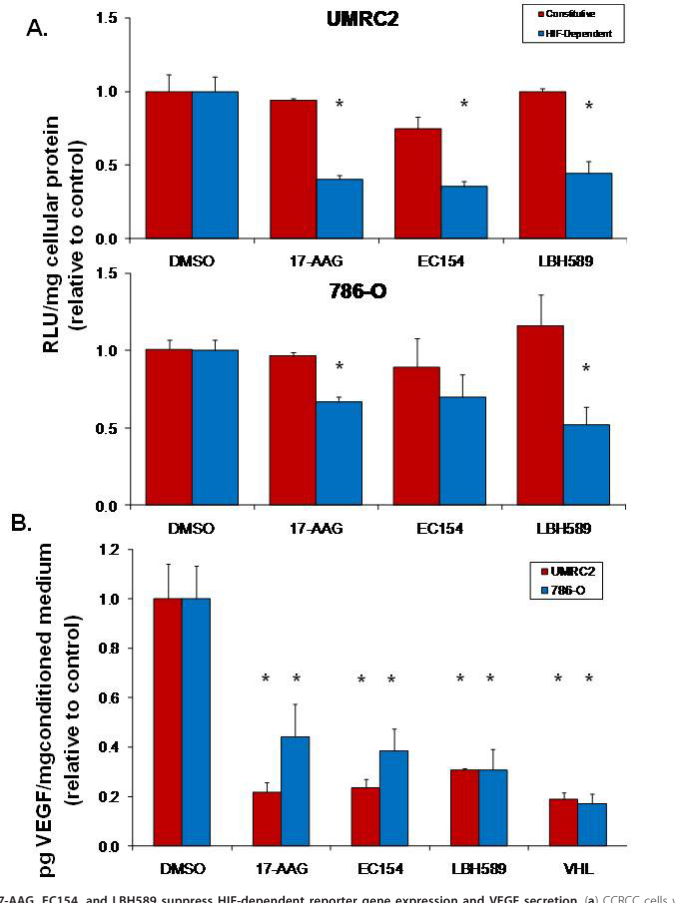
**17-AAG, EC154, and LBH589 suppress HIF-dependent reporter gene expression and VEGF secretion**. (**a**) CCRCC cells were transiently transfected with either a constitutive or VEGF HRE-driven luciferase reporter construct, treated for 16 h with inhibitors, and luciferase enzyme activity determined in whole cell lysates. All drug treatments significantly reduced HIF-dependent reporter gene expression (*) in both cell types with the exception of EC154 in 786-O, as determined by ANOVA and Student's *t-*test (*p *< 0.05). (**b**) CCRCC cells were pre-treated for 4 h with inhibitors in reduced serum DMEM (3% FBS), and incubated for an additional 16 h with freshly prepared treatments in reduced serum medium. Conditioned medium was collected and VEGF levels were analyzed by ELISA. Values were normalized to total protein in conditioned medium and presented relative to controls, with standard deviation. All drug treatments significantly reduced VEGF secretion (*) in both cell types, as determined by ANOVA and Student's *t-*test (*p *< 0.05).

To uncouple some of this complexity and to evaluate the specific effects of these agents upon HIF-dependent transcription, we utilized a HIF-driven luciferase reporter assay system in UMRC2 and 786-O cells. Consistent with our qRT-PCR results, both 17-AAG and EC154 suppressed HIF-driven luciferase expression after 16 h in both cell types, with a greater extent of inhibition observed in UMRC2 (Figure 4a). However, within this model, LBH589 demonstrated comparable effects upon HIF dependent reporter expression in both cell lines, despite eliciting comparatively greater suppression in UMRC2 as determined by qRT-PCR (Figures 2 and 3). Given the variations between HIF-α protein expression and HIF transcriptional activity, we next examined whether the expression of HIF-regulated proteins mirrored the trends of their transcript levels. The HIF signaling pathway regulates numerous pro-tumorigenic pathways including angiogenesis and vasculogenesis [2,64,65] and VEGF is a major contributor of HIF-driven angiogenesis. Hsp90 inhibitors such as 17-AAG decrease HIF-dependent production of CCRCC derived VEGF [66] and LBH589 has been shown to inhibit VEGF signaling in endothelial cells [67]. To further interrogate drug mediated effects upon HIF, we next evaluated the effects of these agents upon VEGF secretion. As shown in Figure 4b, all three drugs significantly reduced secreted VEGF levels in both cell types, congruous with the qRT-PCR results (Figure 3). Interestingly, 17-AAG and EC154 appeared somewhat less effective in reducing intracellular VEGF levels (Additional file 2: Figure S2). The significance of this is not entirely clear given that the secreted protein represents the biologically relevant fraction initiating HIF-mediated angiogenesis. However, this finding illustrates another level of complexity in that intracellular and secreted pools of HIF target proteins may be differentially susceptible to therapeutics.

### Dose dependent efficacy of inhibitors upon VEGF and uPA secretion

**Figure 5:**
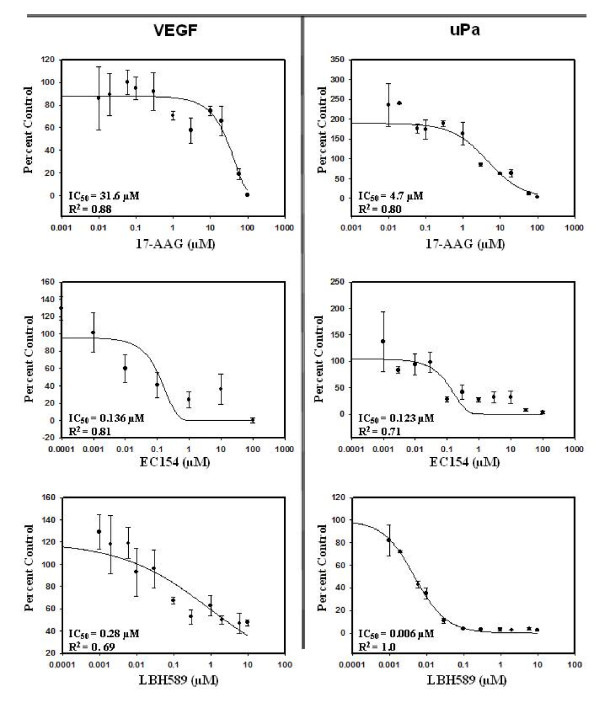
**Ability of agents to suppress VEGF and uPA secretion in UMRC2 cells**. UMRC2 cells were pre-treated for 4 h with the indicated doses of agents in reduced serum as in Figure 4. Conditioned medium was collected and both VEGF and uPA levels were analyzed by ELISA. Values were normalized to total protein in conditioned medium and are presented as percent of cytokine secretion relative to control with standard deviation.

In addition to VEGF, HIF activation promotes secretion of the pro-angiogenic urokinase plasminogen activator (uPA) [68,69]. In conjunction with its receptor uPAR, uPA regulates the plasminogen pathway to stimulate MMP activity and influence bioavailability of many HIF-regulated growth factors [70]. To examine the relative potency of these agents, we evaluated the dose dependent effects of these agents upon secreted VEGF and uPA in UMRC2 cells. Surprisingly, by this metric, EC154 and LBH589 were significantly more potent than 17-AAG (Figure 5), evidenced by their far more robust suppression of both VEGF and uPA (IC_50 _= 0.006-0.28 μM) as compared 17-AAG (14.8-31.6 μM). Interestingly, LBH589 demonstrated the strongest potency against uPA relative to the other agents (IC_50 _= 0.006 μM). Cell viability analysis by MTT assay supports that these drug dependent effects were due specifically to modulation of protein secretion and not a result of decreased cell proliferation (Additional file 3: Figure S3). We previously demonstrated that GA effectively reduced VEGF transcript expression and associated angiogenesis in RCC cells under hypoxic conditions [25]. To confirm that EC154 and LBH589 similarly maintain efficacy under hypoxia, UMCRC2 and 786-O cells were exposed to hypoxia [25,71] for 16 h in either the presence or absence of these agents. As shown (Additional file 4: Figure S4), both EC154 and LBH589 potently suppressed uPA and VEGF secretion under hypoxia.

### Dose dependent effects of inhibitors upon CCRCC cell motility and associated molecular effectors

**Figure 6:**
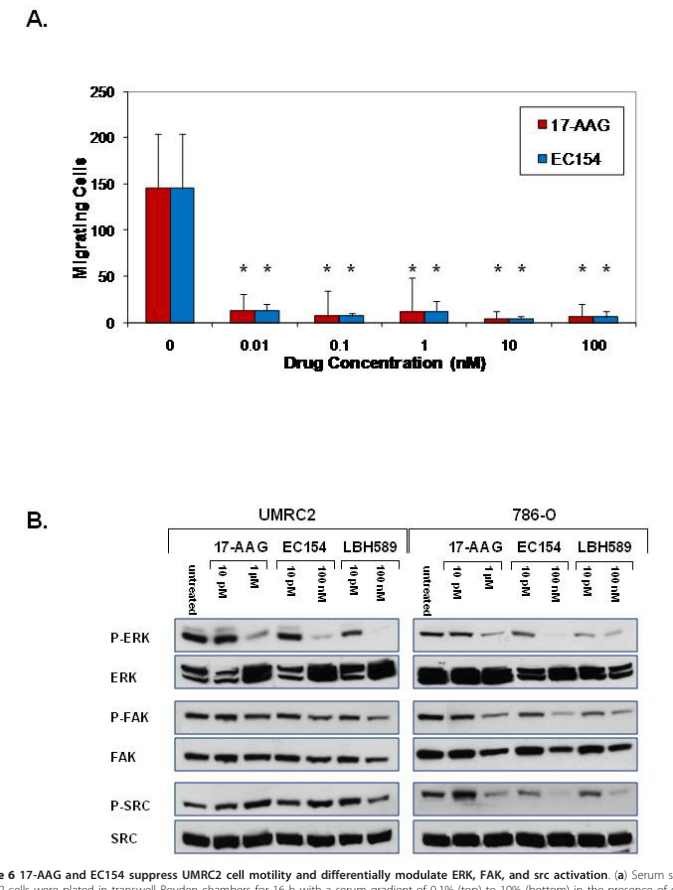
**17-AAG and EC154 suppress UMRC2 cell motility and differentially modulate ERK, FAK, and src activation**. (**a**) Serum starved UMRC2 cells were plated in transwell Boyden chambers for 16 h with a serum gradient of 0.1% (top) to 10% (bottom) in the presence of serially diluted concentrations of the indicated Hsp90 inhibitors. Migrating cells were fixed with 3.7% formaldehyde, stained with Crystal Violet and counted, with standard deviation shown. All drug treatments significantly inhibited migration in both cell types (*), as determined by ANOVA and Student's *t-*test (*p *< 0.05). (**b**) UMRC2 and 786-O cells were treated for 20 h with the indicated doses of inhibitors and phosphorylated and total ERK, FAK, and src were analyzed by SDS-PAGE and Western blot.

The ability of these agents to elicit suppression of VEGF and uPA secretion at doses several log below their suggested optimal dose was intriguing. To explore whether low doses of these agents were able to suppress additional parameters of tumorigenicity, we evaluated their effects upon CCRCC cell motility, a property associated with HIF activity in CCRCC and other models [72-74], and additionally influenced by both VEGF and uPA. As shown in Figure 6a, 17-AAG and EC154 completely ablated UMRC2 cell motility at doses as low as 0.01 nM. In support of this finding, femtomolar levels of geldanamycin derivatives have been previously reported to inhibit invasion in human glioblastoma and myosarcoma cells through a proposed mechanism distinct from degradation of Hsp90 client proteins [75]. To further correlate our migration results with changes in relevant pro-motility signaling mediators, we examined the concentration dependent effects of these agents upon ERK, FAK, and src [76,77]. As shown in Figure 6b, only the high drug concentration of each agent suppressed ERK and FAK activation in UMRC2, the latter only observed with EC154. Src activation was not altered with either 17-AAG or EC154 and was only modestly reduced by the higher dose of LBH589. In contrast, ERK, FAK, and src phosphorylation were more susceptible to drug treatment in 786-O cells, wherein high concentrations of all three drugs reduced phosphorylation of these signaling mediators. Interestingly, low dose 17-AAG appeared to increase src phosphorylation, an effect most profoundly observed in 786-O cells, (10 pM 17-AAG), and not as readily observed with EC154. This 17-AAG mediated upregulation in src phosphorylation correlates with its reported ability to activate src activity in osteoclasts [78]. The drug mediated suppression of cell motility in UMRC2 independent from equivalent suppression of either FAK or src activity suggests that these agents impact upon alternative pro-motility factors. One likely candidate is uPA, given the ability of low drug concentrations to potently impair uPA secretion (Figure 5). Given that uPA regulates cell motility in part via integrin signaling [79], our findings suggest that these agents may impair motility in part via blockade of an uPA-integrin dependent pathway. Other possibilities include changes to the acetylation status of tubulin following inhibition of either Hsp90 or HDACs [80,81].

### Inhibitors potently suppress CCRCC mediated tubule formation but differentially modulate endothelial barrier function

**Figure 7:**
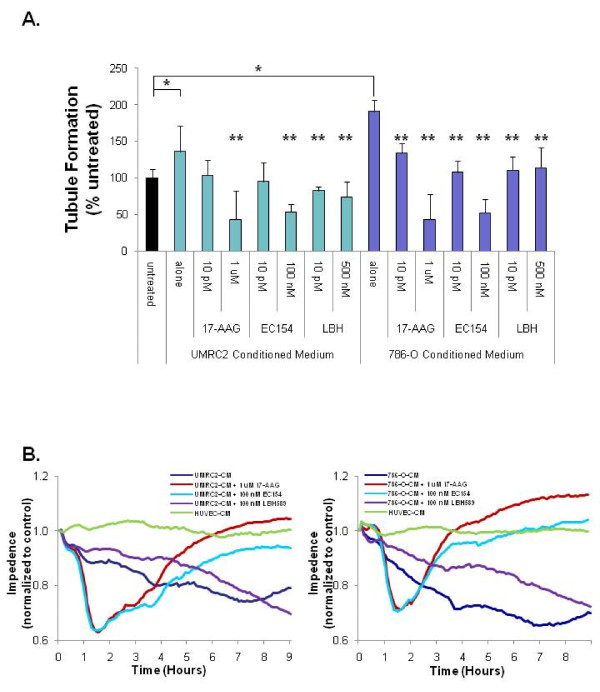
**17-AAG, EC154, and LBH589 suppress CCRCC mediated tubule formation and differentially modulate endothelial barrier function**. **a**) Serum starved (0.1%) HUVEC cells were plated on growth factor reduced Matrigel in the presence of CCRCC conditioned medium (UMRC2 or 786-0) in the presence or absence of 17-AAG, EC154, or LBH589. Tubule formation was imaged at 6 h from 6 replicate wells of a 96-well plate (1 field per well at 40 × magnification), branch points counted, and presented as a percent of untreated control with standard deviation. Conditioned medium from both cell types significantly induced tubule formation (*) as determined by Student's *t-*test (*p *< 0.05). Drug treatments that significantly reversed the effects of conditioned medium are indicated (**) as determined by ANOVA and Student's *t-*test (*p *< 0.05). **b**) Monolayers of HUVEC cells were allowed to reach a minimal TEER plateau and subsequently incubated with conditioned medium from UMCRC2 (left panel) or 786-O cells (right panel) in the presence or absence of 17-AAG, EC154, or LBH589. Conditioned medium from HUVEC cells was used as a negative control. Impedance was measured at 5 min intervals, normalized to levels just prior to the addition of effectors, and presented relative to untreated control. The traces shown represent an average of two replicates per condition.

CCRCC tumors are among the most vascular, demonstrating the clinically important role of HIF in modulating levels of secretory factors that influence the tumor microenvironment. Given that low doses of these inhibitors elicited potent effects upon CCRCC motility, signaling, and secretion of uPA and VEGF, we examined the effects of these agents upon CCRCC mediated angiogenesis. Although previous reports have demonstrated the ability of these agents to inhibit tubule formation initiated by a single specific growth factor [31,67], tumors promote angiogenesis via secretion of a complex mixture of growth factors. We therefore determined the relative ability of these agents to inhibit tubule formation when challenged by exposure to the physiologically relevant secreted milieu derived from CCRCC cells. As shown in Figure 7, conditioned medium derived from both UMRC2 and 786-O cells stimulated HUVEC tubule formation within 6 h, an effect attenuated with the addition of each of the three drugs. Importantly, low dose treatment of all three drugs reduced tubule formation to levels comparable to the untreated control, and high dose treatment of the ATP Hsp90 inhibitors 17-AAG and EC154 further inhibited tubule formation to levels below untreated controls.

A complementary critical component of vessel function is permeability. Paracrine signals from the tumor promote the weakening of endothelial cell junctions. As a result of this diminished barrier function, the tumor vasculature becomes characteristically disordered, permeable, and leaky. This enhanced permeability promotes the ability of cancer cells to breach the vasculature in a process termed transendothelial migration (TEM) [82]. VEGF is among the best studied mediators of endothelial paracellular permeability [83]. Although CCRCC tumors are highly angiogenic, no reports have investigated the cumulative effects of CCRCC secreted factors upon endothelial barrier function, nor have any of the agents herein been evaluated for their capacity to counteract paracrine tumor factors that breach endothelial cell integrity. To examine endothelial barrier function upon challenge with CCRCC conditioned medium, and the ability of the inhibitors to counteract potential effects of this exposure, we utilized electric cell-substrate impedance sensing (ECIS). As shown in Figure 7b, conditioned medium from both UMRC2 and 786-O cells dramatically reduced endothelial cell barrier function. This effect was not replicated by conditioned medium from HUVECs, validating CCRCC derived specific effects. Moreover, the effect of CCRCC conditioned medium was greater than that of VEGF alone (Additional file 5: Figure S5) supporting the notion that tumor cells modulate endothelial cell integrity via a complex mixture of effectors. Importantly, both 17-AAG and EC154 were able to restore barrier function in HUVECs challenged with CCRCC conditioned medium. Although 17-AAG has been previously demonstrated to restore barrier function upon exposure to a single cytokine [84,85], this is the first study to highlight its ability to maintain endothelial cell integrity upon exposure to a complex mixture of tumor derived paracrine factors. Strikingly, a similar recovery effect was not observed with LBH589. That LBH589 did not restore endothelial barrier function in a similar manner is unexpected and suggests the possibility a non-Hsp90 mediated effect.

## Discussion

Given the well established role of HIF in CCRCC, and its seemingly universal involvement in the progression of diverse solid tumors, it is critically important to evaluate the HIF targeting ability of next-generation Hsp90 inhibitors. However, surprisingly few studies have examined this particular aspect of these agents. The results from this study indicate that standard endpoints for HIF function may be less informative than previously indicated, and that the apparent complexity of inhibitor effects requires a more functionally based and integrative approach. One example of this complexity is demonstrated by our findings that although all three compounds significantly reduced HIF-1α protein levels, consistent with previous reports [25-27,45], 17-AAG and EC154 did not appreciably diminish HIF-2α levels, and surprisingly, LBH589 increased HIF-2α expression. To our knowledge, this is the first report to examine the effect of an HDAC inhibitor on HIF-2α levels, and more significantly, to demonstrate an HDAC dependent increase in protein expression. It remains unclear whether the mechanism of LBH589 mediated increase of HIF-2α protein may be due to changes in protein degradation or synthesis. Further, the requirement of RACK1 for Hsp90-inhibition mediated HIF-2α degradation has not been established. The differential response of the HIF isoforms is not without precedent, in that isoform specificity for HIF-1α and HIF-2α has been demonstrated during oxygen and VHL independent destruction pathways [86,87]. However, of greater relevance to this study is that the LBH-589 mediated increase in HIF-2α expression did not elicit a comparable increase in HIF-2α activity.

Our findings reinforce the theme that HIF expression and activity may be uncoupled events, a notion previously observed following treatment with proteasomal inhibitors [59]. First, although LBH589 increased HIF-2 expression, all other parameters of HIF activity support an overall suppressive effect. Given that HIF activity is regulated by acetylation, it is very possible that the increase in HIF acetylation by LBH-589 is inhibitory. Further, increased acetylation has also been shown to inhibit Hsp90 chaperone activity [44], which may further suppress HIF-2 activity. Second, all three drugs diminished HIF-1α and HIF-2α dependent transcription within 2 h of treatment, prior to decreases in protein levels [25,88], and data not shown. Although EC154 and 17-AAG demonstrated approximately equivalent kinetics of suppression, exposure of cells to low concentrations of EC154 (10-fold lower levels than the experimental 100 nM) did not elicit detectable increases in src phosphorylation, indicating an improved profile compared with 17-AAG.

Our data highlight the complexity of drug targeting, in that the drug mediated suppression of HIF target genes varied dynamically with time and dose, and differential sensitivity was observed in a transcript and cell context dependent manner. This complexity is not surprising given that additional modifiers may contribute to the net effects of Hsp90 inhibition. For example, NF-kB promotes HIF-1α transactivation in an acetylation sensitive manner [89] and coordinates suppressive effects upon NF-kB, and Hsp90 may contribute to the robust inhibition of HIF activity observed with LBH589. Moreover, HDAC inhibition at early time points has also been demonstrated to disrupt the p300:HIF complex, a critical component of HIF transactivation, resulting in decreased HIF transcriptional activity [88]. Adding to this complexity, HIF directly interacts with, and is regulated by, HDACs [90] illustrating several nodal points by which Hsp90 and HDAC inhibitors may distinctly impact upon HIF function.

To more comprehensively evaluate the relative efficacy of these agents against specific metrics, we examined key anticancer properties beyond HIF transcription. Sub-nanomolar concentrations of 17-AAG and EC154 profoundly suppressed CCRCC cell motility, whereas higher concentrations were required to diminish CCRCC-mediated HUVEC tubule formation. Although LBH589 demonstrated the most potent suppression of both *VEGF *transcription and secreted protein, it only modestly prevented tubule formation in 786-O. Our tubule formation results support the notion that Hsp90 inhibiting agents, in part via their suppressive effects upon HIF, may be utilized clinically to reduce the vascularity associated with CCRCC and other solid tumors [31,67]. Although LBH589 did not restore barrier function, another report demonstrates that this agent, in combination with rapamycin in a preclinical model, was effective in reducing CCRCC angiogenesis [15]. A recent report found that pre-incubation with the HDAC6 inhibitor tubacin prevents thrombin induced barrier damage [91], suggesting that the effects of HDAC inhibition upon vessel permeability could vary greatly depending upon a variety of factors. Therefore, the ECIS assay may be a useful approach to more fully interrogate the ability of LBH589, and other HDACIs, to restore endothelial cell function alone and in combination with currently utilized anticancer agents. The ability of these agents to preserve endothelial cell integrity could have significant effects on clinical outcome and warrants further attention. Clinically, vascular permeability can promote tumor cell TEM and metastasis [82] and poses a further challenge in diminishing the delivery and efficacy of chemo- and radio-therapies [92]. Our results suggest that these agents may have utility in 'normalizing' the tumor vasculature, an effect that improves chemo- and radio-therapy [92]. Although not formally demonstrated for Hsp90 inhibitors, 17-AAG and radicicol have been shown to improve endothelial barrier formation and rescue barrier in the presence of damaging agents including thrombin, VEGF, and phorbal ester [84,85]. In addition, we previously demonstrated that both GA and 17-AAG potentiate the radiation response of cervical tumor cells *in vitro *and *in vivo *[29]. Our data suggest that EC154 exhibits a similar capacity as 17-AAG in terms of its ability to restore barrier function, and may therefore represent a viable clinical candidate.

## Conclusions

In sum, our transcriptional results indicate that Hsp90 inhibition may affect HIF-dependent gene transcription through at least two mechanisms; by direct inhibition of transcriptional activity at early time points, and by promotion of HIF-1α degradation at later time points. This further highlights the complexity of Hsp90 inhibitor effects and weakens the notion that variations of HIF-α isoform stability is a primary reason for the clinical failure of these agents [34]. Importantly, our results also highlight that HIF expression is not necessarily a reliable surrogate for interrogating the HIF targeting efficacy of Hsp90 inhibitors. This is a particularly salient finding in light of recent efforts to improve *in vivo *and intratumoral imaging of Hsp90 client proteins as a surrogate for monitoring inhibitor activity [93-97]. Moreover, our results further indicate that levels of HIF regulated cytokines such as VEGF may also be unreliable surrogates for HIF activity, demonstrated by the observed differential effects of Hsp90 inhibition upon intracellular and secreted VEGF. This finding further highlights the challenge in choosing appropriate biomarkers, in that the histochemical analysis of tumor tissues for VEGF expression would indicate less robust drug dependent effects compared with an analysis of secreted levels of the same protein. Our findings highlight the need to integrate multiple, diverse, and functional endpoints to more appropriately discern the effects of Hsp90 inhibition upon HIF function. With more than a dozen new Hsp90 inhibitors currently under clinical evaluation [98], there is a critical need to establish relevant and reliable surrogates and biomarkers for evidence of Hsp90 inhibition. The identification of these readouts and their subsequent incorporation into clinical trials will provide useful indicators of positive response. Our findings offer useful metrics and considerations to guide the preclinical and clinical evaluation of conventional and novel Hsp90 inhibitors.

## Competing interests

This work was funded in part by a commercial grant from Biogen Idec.

## Authors' contributions

JEB participated in the design of the study, performed immunoblots, immunoassays, cell migration, tubule formation, cell viability and ECIS experiments, statistical analysis, and drafted the manuscript. SP carried out the qRT-PCR studies, lentiviral infections, and reporter assays. UG carried out studies pertinent to the activity status of pro-motility effector proteins. MWH participated in the cell migration studies. SW and KA participated in the design, execution, and analysis of the ECIS experiments. KL participated in the design and coordination for the study and provided EC154. JSI conceived of the study, participated in its design and coordination, and edited the manuscript. All authors read and approved the final manuscript.

## Pre-publication history

The pre-publication history for this paper can be accessed here:

http://www.biomedcentral.com/1471-2407/11/520/prepub

## Supplementary Material

Additional file 1 **Figure S1**. Dose dependent effects of 17-AAG, EC154, and LBH589 upon HIF-dependent gene transcription in 786-O. 786-O cells were treated for 16 h with the indicated concentrations of inhibitors, total mRNA was isolated and HIF-α regulated genes analyzed by QRT-PCR. Values were normalized to GAPDH and are presented relative to control, with standard deviation. Stably transfected VHL replaced cells were used as a control condition for HIF suppression. Click here for file

Additional file 2 **Figure S2**. Effects of 17-AAG, EC154, and LBH589 upon intracellular VEGF expression in CCRCC cells. CCRCC cells (786-O and UMRC2) were pre-treated for 4 h (1 μM 17-AAG, 100 nM EC154, 100 nM LBH589) in reduced serum DMEM (3% FBS). Cells were then re-incubated for an additional 16 h with freshly prepared treatments in reduced serum medium. Whole cell lysate was collected and VEGF levels were determined by ELISA. Values are normalized to total cellular protein and presented as a percent of DMSO treated control with standard deviation. All drug treatments significantly reduced HIF-dependent reporter gene expression (*) in both cell types with the exception of EC154 in 786-O, as determined by ANOVA and Student's *t-*test (*p *< 0.05). Click here for file

Additional file 3 **Figure S3**. Administration of 17-AAG, EC154, and LBH589 does not affect CCRCC viability within 16 h. CCRCC cells were incubated for 16 h with vehicle or the indicated agents and cell viability was determined by MTT assay, with data presented as a percent of control cells, with standard deviation. Click here for file

Additional file 4 **Figure S4**. Suppression of VEGF and uPa secretion by EC154 and LBH589 in CCRCC cells under hypoxia. CCRCC cells were pre-treated for 4 h with inhibitors in reduced serum DMEM (3% FBS), and incubated for an additional 16 h with freshly prepared treatments in reduced serum medium at 1% O_2_. Conditioned medium was collected and VEGF and uPa levels were analyzed by ELISA. Values were normalized to total protein in conditioned medium and presented relative to controls, with standard deviation. Click here for file

Additional file 5 **Figure S5**. VEGF elicits a modest breach of endothelial integrity, which is rescued by 17-AAG. Monolayers of HUVEC cells were allowed to reach a minimal TEER plateau and then incubated with VEGF (50 ng/mL) in the presence or absence of 17-AAG (1 μM). Impedance was measured at 5 min intervals, normalized to levels just prior to the addition of effectors, and presented relative to untreated control. The traces shown represent an average of two replicates per condition. Click here for file
