# Supplementary figures and images for: Comparative analysis of novel and conventional Hsp90 inhibitors on HIF activity and angiogenic potential in clear cell renal cell carcinoma: implications for clinical evaluation
Source: BMC Cancer. 2011 Dec 15;11:520. doi: 10.1186/1471-2407-11-520 (PMC3259130; doi:10.1186/1471-2407-11-520)

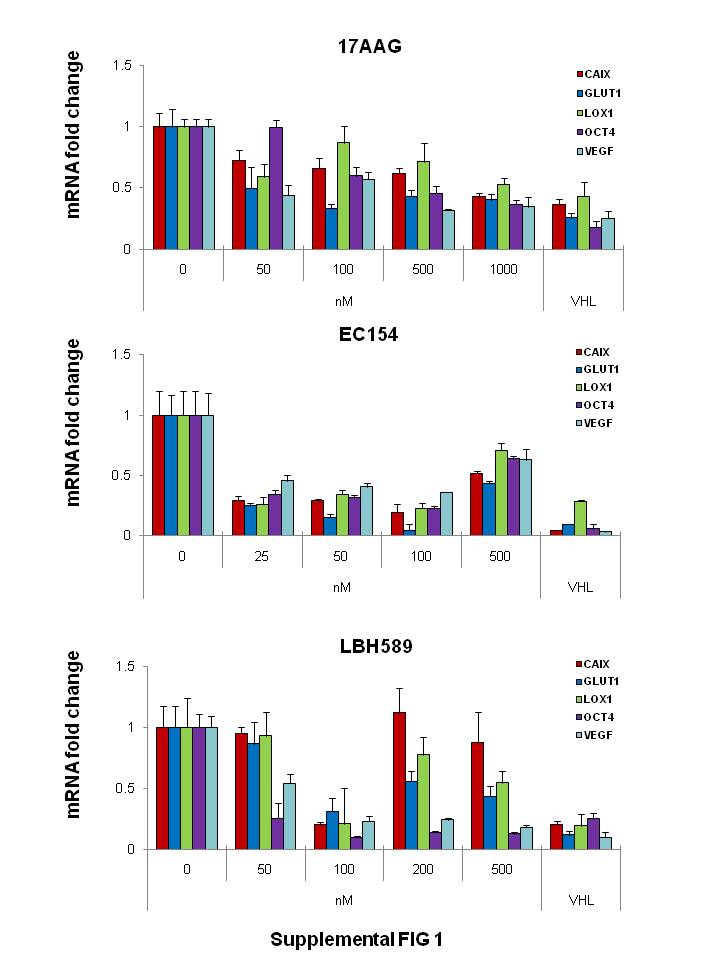

Supplement: Additional file 1 — Figure S1. Dose dependent effects of 17-AAG, EC154, and LBH589 upon HIF-dependent gene transcription in 786-O. 786-O cells were treated for 16 h with the indicated concentrations of inhibitors, total mRNA was isolated and HIF-α regulated genes analyzed by QRT-PCR. Values were normalized to GAPDH and are presented relative to control, with standard deviation. Stably transfected VHL replaced cells were used as a control condition for HIF suppression. [file 1471-2407-11-520-S1.JPEG]

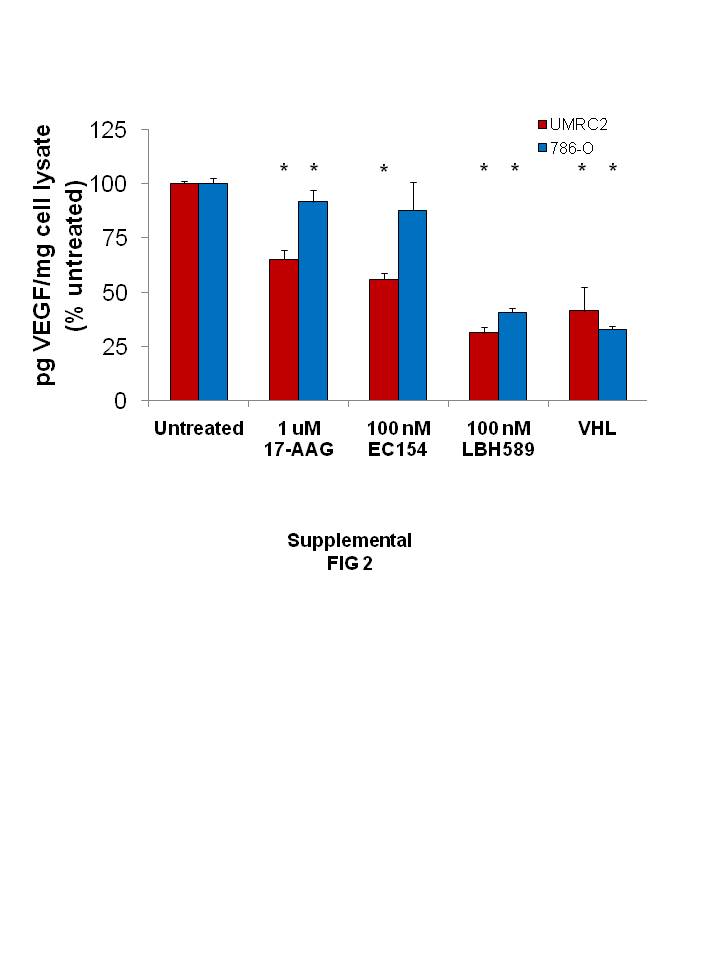

Supplement: Additional file 2 — Figure S2. Effects of 17-AAG, EC154, and LBH589 upon intracellular VEGF expression in CCRCC cells. CCRCC cells (786-O and UMRC2) were pre-treated for 4 h (1 μM 17-AAG, 100 nM EC154, 100 nM LBH589) in reduced serum DMEM (3% FBS). Cells were then re-incubated for an additional 16 h with freshly prepared treatments in reduced serum medium. Whole cell lysate was collected and VEGF levels were determined by ELISA. Values are normalized to total cellular protein and presented as a percent of DMSO treated control with standard deviation. All drug treatments significantly reduced HIF-dependent reporter gene expression (*) in both cell types with the exception of EC154 in 786-O, as determined by ANOVA and Student's t-test (p < 0.05). [file 1471-2407-11-520-S2.JPEG]

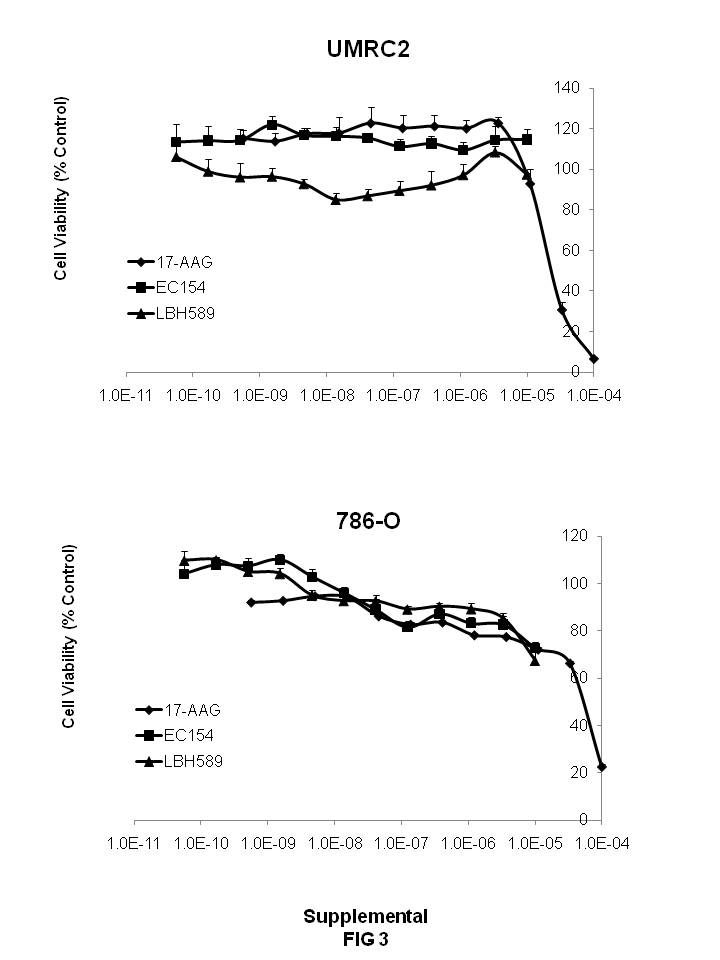

Supplement: Additional file 3 — Figure S3. Administration of 17-AAG, EC154, and LBH589 does not affect CCRCC viability within 16 h. CCRCC cells were incubated for 16 h with vehicle or the indicated agents and cell viability was determined by MTT assay, with data presented as a percent of control cells, with standard deviation. [file 1471-2407-11-520-S3.JPEG]

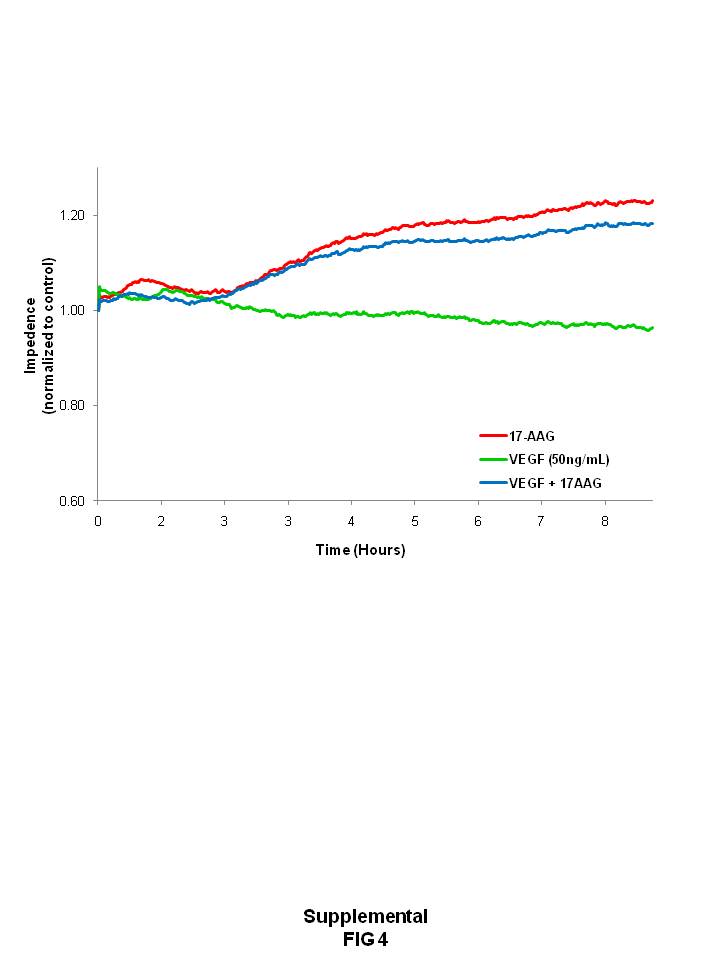

Supplement: Additional file 4 — Figure S4. Suppression of VEGF and uPa secretion by EC154 and LBH589 in CCRCC cells under hypoxia. CCRCC cells were pre-treated for 4 h with inhibitors in reduced serum DMEM (3% FBS), and incubated for an additional 16 h with freshly prepared treatments in reduced serum medium at 1% O2. Conditioned medium was collected and VEGF and uPa levels were analyzed by ELISA. Values were normalized to total protein in conditioned medium and presented relative to controls, with standard deviation. [file 1471-2407-11-520-S4.JPEG]

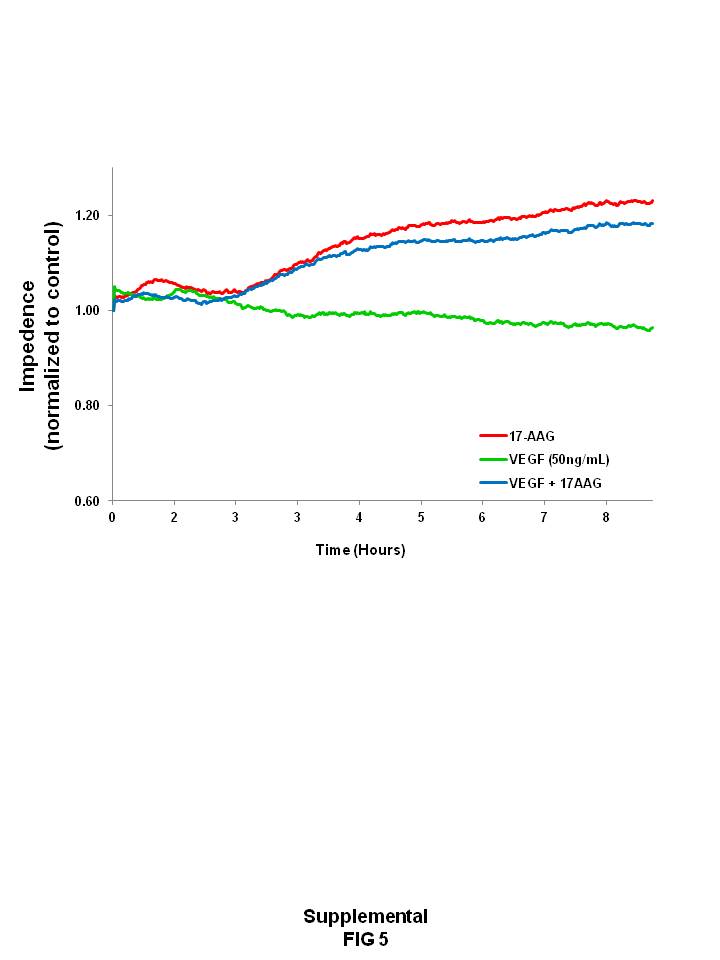

Supplement: Additional file 5 — Figure S5. VEGF elicits a modest breach of endothelial integrity, which is rescued by 17-AAG. Monolayers of HUVEC cells were allowed to reach a minimal TEER plateau and then incubated with VEGF (50 ng/mL) in the presence or absence of 17-AAG (1 μM). Impedance was measured at 5 min intervals, normalized to levels just prior to the addition of effectors, and presented relative to untreated control. The traces shown represent an average of two replicates per condition. [file 1471-2407-11-520-S5.JPEG]
